# An old approach to a novel problem: effect of combined balance therapy on virtual reality induced motion sickness: a randomized, placebo controlled, double-blinded study

**DOI:** 10.1186/s12909-024-05152-4

**Published:** 2024-02-19

**Authors:** Kurul Ramazan, Altuntas Yasin Devran, Ogun Nur Muhammed

**Affiliations:** 1 Department of Physical Therapy and Rehabilitation, Faculty of Health Sciences, Bolu Abant Izzet Baysal University, Bolu, Turkey; 2 Department of Neurology, Faculty of Medicine, Bolu Abant Izzet Baysal University, Bolu, Turkey

**Keywords:** Cyber sickness, Education, Exercise, Virtual reality

## Abstract

**Background:**

The objective of this study was to investigate the impact of a rehabilitation program aimed at addressing vestibular and proprioceptive deficits, which are believed to underlie the pathophysiology of motion sickness.

**Methods:**

A total of 121 medical students with motion sickness participated in this study and were randomly divided into intervention (*n* = 60) and placebo control (*n* = 61) groups. The intervention group underwent combined balance, proprioception, and vestibular training three times a week for 4 weeks, while the control group received placebo training. The study assessed various measurements, including the Virtual reality sickness questionnaire (VRSQ), tolerance duration, enjoyment level measured by VAS, stability levels using Biodex, and balance with the Flamingo balance test (FBT). All measurements were conducted both at baseline and 4 weeks later.

**Results:**

There was no significant difference in pre-test scores between the intervention and control groups, suggesting a similar baseline in both groups (*p* > 0.05). The results showed a significant improvement in VRSQ, tolerance duration, VAS, Biodex, and FBT scores in the intervention group (*p* < 0.05). While, the control group showed a significant increase only in VAS scores after 4 weeks of training (*p* < 0.05). A statistically significant improvement was found between the groups for VRSQ (*p* < 0.001), tolerance duration (*p* < 0.001), VAS (*p* < 0.001), Biodex (*p* = 0.015), and FBT scores (*p* < 0.05), in favor of the intervention group.

**Conclusions:**

A combined balance training program for motion sickness proves to be effective in reducing motion sickness symptoms, enhancing user enjoyment, and extending the usage duration of virtual reality devices while improving balance and stability. In contrast, placebo training did not alter motion sickness levels. These findings offer valuable insights for expanding the usage of virtual reality, making it accessible to a broader population.

## Introduction

Virtual reality (VR) provides an immersive three-dimensional experience capable of replicating reality with remarkable fidelity or transporting users to entirely fictional environments. In both cases, users become fully immersed in this virtual realm, where they feel an authentic presence and interact seamlessly with their surroundings [1]. Ongoing developments and research in virtual reality technologies have expanded their applications across various disciplines, including higher education, medicine, video gaming, healthcare, and social sciences such as history [1, 2].

The utilization of virtual reality within health professions education is on the rise and is becoming a significant component in competency development. Immersive technologies can provide similar educational benefits to traditional learning methods [3]. One of the main reasons why virtual reality is preferred in the field of medical education is that it offers interaction environments that allow cadaver dissection and direct manipulation and can provide many different scenarios with personalized modifications [4]. Additionally, VR allows students and newcomers to engage with intricate scenarios in a secure and controlled setting, offering the opportunity for iterative practice without the wear and tear associated with costly physical simulators. Employing VR technology for training purposes can significantly reduce error rates, enhance the learning process, save time, and lower costs [5]. However, the integration of such technology into higher education has been gradual, impeded by various technological barriers, and there seems to be a dearth of effective sharing of innovative practices and successful implementations [6].

Prolonged exposure to virtual environments and simulators has been associated with adverse effects, with approximately 30% of users experiencing nausea and up to 40% reporting eyestrain, which can substantially impede user participation [7, 8]. These adverse effects have been described in the literature using several terms, most commonly referred to as ‘cybersickness’ or ‘motion sickness’ (MS) [9]. Motion sickness occurs during movements that do not correlate with bodily motions and creates sensory conflict or sensory mismatch between the actual sensory information, encompassing kinaesthetic inputs, vestibular, visual, and sensory patterns derived from the virtual environment [10, 11], such as those encountered during airplane or boat travels [12].

Predicting the occurrence of MS in a user is exceedingly challenging due to the multitude of factors, encompassing both technological and individual aspects, that contribute to its causation [13]. The pathophysiology of MS is built around sensory conflict and neural storage theories, which suggest that alterations in visual stimuli can give rise to sensory conflicts among proprioception and vestibular inputs (vestibulo-ocular/vestibulo-spinal reflexes), consequently eliciting varying degrees of visually induced MS [14–16]. Despite ongoing advancements in related technologies and recent innovations, adverse effects induced by virtual reality in simulator and virtual environments continue to be reported in recent literature. For instance, a recent publication reported a mean dropout rate of 15.6% based on data collected from 44 empirical studies on MS and the impact of VR content when using a head-mounted display [17]. Motion sickness symptoms are categorized into two subgroups: oculomotor and disorientation [18]. It is believed that ocular symptoms are related to vestibular connections, while general symptoms are thought to be associated with the somatosensory system and proprioceptive sources [15, 19]. The apparent sense of self-motion is primarily controlled by the visual system. Motion sickness in this context is often referred to as visually triggered motion sickness and causes additional oculomotor problems [20]. Therefore, in the VR environment users may suffer from motion illusions; a false motion sense that caused due to visual stimulation without actual physical movement. This phenomenon can lead to cases of motion sickness in virtual reality [21]. Visual, vestibular, and somatosensory stimuli are often integrated for self-motion perception, but in VR these stimuli can conflict with each other and cause motion sickness [22, 23]. This high incidence of side effects is a major obstacle to the use of this technology in education. Therefore, the aim of this study was to investigate the effects of a combined training program focusing on both somatosensory and proprioceptive training on virtual reality induced motion sickness symptoms in healthy medical students.

## Methods

One hundred twenty-one medical students who accepted and fulfilled the inclusion criteria were recruited for this study. This randomized controlled, double-blinded study was performed in line with the Helsinki Declaration with permission from the local ethics committee of xxxx University (Clinical Research Ethics Committee 2022/299–503) (clinicaltrials.gov ID: NCT06056622 28/09/2023) and conducted in the department of physical therapy and rehabilitation. Inclusion criteria consisted of presenting motion sickness symptoms with a minimum score of 30 on the Virtual reality sickness questionnaire (VRSQ) and a stereoacuity score of 3552 arc/s on the Titmus Fly Test. Stereopsis was measured at close range (40 cm) using the Stereo Fly Test (Stereo Optical Company Inc., Chicago, IL, USA) with the Titmus graded circle stereo test. This test consists of nine-panel graded circles with stimulus disparity ranging from 800 to 40 arc/seconds. Each panel contained four contoured circles, only one of which had a crossed disparity. Subjects were instructed to wear polarized glasses to identify the circle that appeared to pop out of the plane. Exclusion criteria encompassed individuals who wore glasses, had a diagnosis of strabismus, had a history of vertigo, had partial or total vision loss, and had previous exposure to head-mounted virtual reality devices.

The sample size calculation was conducted using G*Power (Universität Düsseldorf, Kiel, Germany). The effect size was determined based on the results of eligibility testing VRSQ scores (effect size, d = 0.56). To achieve a significance level (α) of less than 0.05 and a power (1-β) of 80%, a total of 52 participants were needed for each group. The participants were randomly assigned in a parallel design (1:1) as intervention (*n* = 60) and control (*n* = 61) groups, with stratification based on sex, age, Purdue Spatial Visualization Test scores (PSVT-R) [24], lateralization scores, and Titmus stereoacuity scores. This stratified randomization was conducted by a researcher (M.N.O.) using a web-based randomization system. Laterality and PSVT-R assessments were used for randomization due to the potential impact of differences in spatial abilities on users’ interaction with the virtual environment, as well as stereoacuity necessary for 3D and depth perception.

Testing initiated with the panel displaying the largest disparity and progressed sequentially to the next panel until the patient made a false selection. In the event of a false selection, the previous panel was retested. If subjects were unable to identify the stimulus with the highest disparity (800 arcseconds), the Stereo Fly diagram was presented, and participants had to pinch the Fly wings to reach 3500 arcseconds.

To achieve a double-blinded design, outcome assessments were conducted by a researcher who was unaware of the group allocation (R.K.), and participants in the control group received placebo training sessions.

### Interventions

Multisensory stimulation with active movement, progressive Cawthorne-Cooksey exercises, and balance exercises with external perturbation were used in the intervention group, while the control group received placebo sessions. Both groups received allocated sessions three times a week for 4 weeks. All exercises were performed under the supervision of a researcher (Y.D.A.).

The multisensory stimulation with active movement treatment was divided into two parts. The first part consists of exercises on a wobble board, including squats, passing a ball between hands and to another person, single-leg stance, and gently pushing each other off balance. The second part consisted of exercises performed on a soft mat: jumping from a small box and landing with both knees bent, passing the ball to a teammate during a two-leg jump, performing a two-leg jump while rotating the trunk 90 degrees, and jumping from a small box onto a soft mat on one leg. Each of these exercises was repeated 10 times during each session.

Cawthorne-Cooksey exercises were administered to stimulate the vestibular system. The exercises consisted of repetitive, progressively more challenging eye, head, and trunk movements, incorporating flexion, extension, and rotation patterns. The exercises were performed in both sitting and upright positions, with eyes open and closed. Each exercise consisted of 10 repetitions, and each set lasted for 10 minutes.

Balance exercises were conducted while participants stood with their feet shoulder-width apart on both hard and soft surfaces, while participants alternated between having their eyes open and closed. In each exercise step, participants were initially informed of the direction of external perturbation, and then perturbations were repeated without prior notification.

Participants in the control group received placebo treatment sessions. These placebo sessions consist of a 10-minute exposure to a visual evoked potential (VEP) measurement screen while holding a mouse. Participants were instructed to click the mouse when the screen changed color. This was done while sitting on an arm-supported chair. The rationale for selecting this placebo treatment protocol was based on the premise that VEP measurements do not typically trigger vestibulo-ocular and vestibulo-spinal reflexes, as color changes occur across the entire screen without necessitating visual focus or balance-related activities during the test.

### Outcome measurements

After obtaining informed consent, demographic information about the patients was collected. Participants’ balance was measured using the Flamingo Balance Test (FBT), and stability was assessed using the Biodex Balance System (Biodex Medical Systems, Shirley, NY). Symptoms related to virtual reality were measured using the VRSQ. The participants’ total usage time before discontinuing the use of VR was recorded in minutes, and they were asked to rate their general tolerance on a visual analog scale (VAS). All measurements were conducted at baseline and again in the fourth week after the intervention was completed. Both pre-and post-test measurements were taken after the participants experienced a 15-minute roller coaster VR session. Roller coaster game was selected due to its ability to induce a high level of motion sickness, attributed to its rapid ascents and descents, rotational motion, and acceleration-deceleration elements [25]. To eliminate any potential learning effect, the track used in the pretest was mirrored.

### VRSQ

Motion sickness symptoms were assessed using a 9-question Likert scale. Participants were instructed to indicate the extent to which each of the following symptoms affected them, ranging from 0 (none) to 3 (severe). The total score for the test amounts to 100, with scores derived from two subsections: oculomotor symptoms and discomfort. The VRSQ is a reliable, valid, and easy-to-administer questionnaire due to its in fewer items and sensitive to diagnosing motion sickness [26].

### Visual analog scale

The participants were instructed to indicate the level of their enjoyment on a 100-mm horizontal line. The intensity of the experience was determined by measuring the segment between the points marked by the individual, ranging from 0 (unenjoyable) to 10 (the most enjoyable experience).

### Biodex balance system

The test records angular displacement of the platform in both the anteroposterior (AP) and mediolateral (ML) axes, providing a measure of postural stability and balance. The ML stability index (MLSI) is derived from angular displacement in the frontal plane on a circular platform, while the AP stability index (APSI) is obtained from angular displacement in the sagittal plane on the same platform. The overall stability index (OSI) is calculated as a composite of the APSI and MLSI. During the measurements, participants were instructed to stand on the platform, either on 1 foot or both feet, as per the protocol, and maintain a static posture. The platform setting was static, the trial time was 20 seconds and the test was performed 3 times, and the best score was used for data analysis [27].

### Flamingo balance test

The test was used to assess participants’ static balance, with trials performed both with eyes closed and eyes open. Each participant was instructed to position the ankle of their untested leg behind the knee of the tested leg while standing on a single leg for 1 minute. The number of visible body sways was recorded for the test duration [28]. The test has high sensitivity and good reliability for determining balance problems in healthy populations especially young adults and teenagers [28, 29].

### Statistical analysis

Numeric variables in the study are presented as the mean ± standard deviation (X ± SD) or as numbers and percentages (n, %). Paired-sample t-test were used to compare pretreatment and posttreatment differences. Group comparisons were conducted by using the ANCOVA test. Cohen’s d formula was used for effect size calculations. To explore the relationship between VRSQ and Titmus scores, Spearman correlation tests were conducted. The statistical significance threshold for all tests was set at *p* < 0.05. Statistical analysis of the data was carried out using SPSS 22.0 for Windows.

## Results

**Fig. 1:**
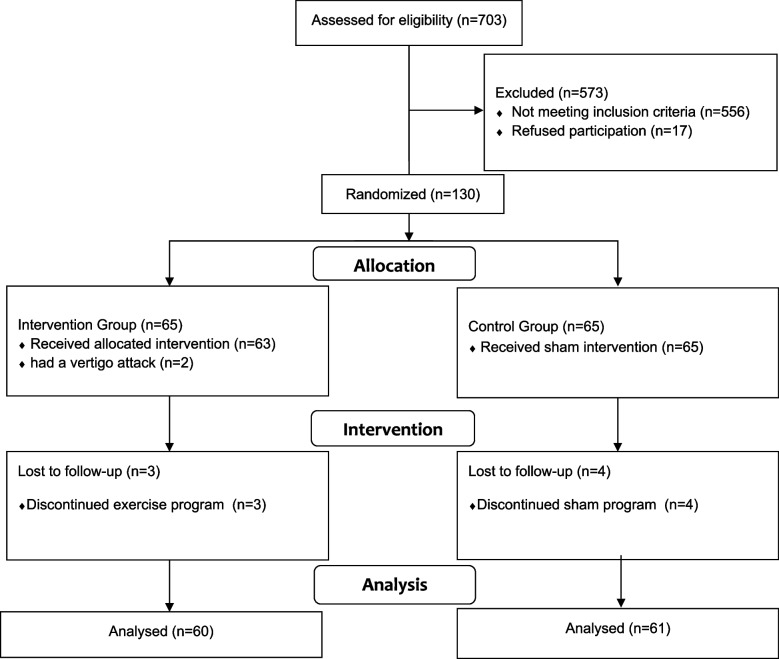
Flowchart of study

A total of 130 medical students were included between December 2022 and April 2023, 121 of whom completed this study (Fig. 1). Among these participants, 44 (73.3%) were female, 16 (26.7%) were male in the intervention group, and 45 (73.8%) were female, with 16 (26.2%) being male in the control group. The normal distribution of data across all outcome assessments for both groups was assessed using the Shapiro–Wilk test. The results revealed that all parameters displayed a normal distribution (*p* > 0.05). No significant differences were found between the groups in terms of sex (*p* = 0.957), age (*p* = 0.530), Titmus stereoacuity scores (*p* = 0.173), lateralization scores (*p* = 0.606), or Purdue Spatial Visualization Test scores (*p* = 0.539) (Table 1).

**Table 1:** Descriptive statistics of participants

	Intervention (*n* = 60)	Placebo control (*n* = 61)		
	X ± SD	X ± SD	t	*p*
**Age (year)**	19.98 ± 0.79	19.88 ± 0.91	−0.630	0.530
**PSVT-R**	20.51 ± 4.24	21.01 ± 4.66	0.616	0.539
**Laterality**	19.78 ± 7.69	20.50 ± 7.72	0.517	0.606
**BMI**	22.43 ± 2.79	22.17 ± 3.64	−0.443	0.658

*PSVT-R* Purdue Spatial Visualization Test Rotations: *BMI* Body Mass Index: t independent samples t test, *p* < 0.05

A comparison of baseline parameters between the groups demonstrated no significant differences at the outset of the study for tolerance time (*p* = 0.622), VRSQ total (*p* = 0.307), VAS (*p* = 0.493), OSI (*p* = 0.241), and FBT scores (*p* = 0.487).

In this study, the primary outcome measurement was the VRSQ score values of the groups. The effect size was calculated using Cohen’s d formula and was determined to be 0.18 for the pre-test results. The study exhibited a power level of 99%, as determined by post hoc power analysis, and effect size was found as 0.465.

In the intervention group, the within-group pre-posttest differences showed a significant decrease in VRSQ total, oculomotor, discomfort, OSI, and FBT scores, along with an increase in tolerance and VAS scores (*p* < 0.05). For the placebo control group, a significant increase was found only in the VAS scores (*p* = 0.031). While, there were no significant differences in the other outcome measures (*p* > 0.05) (Table 2).

**Table 2:** Results of pre- and post-intervention scores within groups

	Intervention (*n* = 60)			Placebo control (*n* = 61)		
	Pre-test	Post-test	*p*	Pre-test	Post-test	*p*
**VRSQ Total**	80.65 ± 10.01	46.23 ± 16.79	**< 0,001**	78.54 ± 12.43	74.24 ± 13.69	0.067
**VRSQ Oculomotor**	35.46 ± 4.83	21.15 ± 7.98	**< 0,001**	34.57 ± 5.93	33.82 ± 6.17	0.494
**VRSQ Discomfort**	45.18 ± 5.70	25.08 ± 8.93	**< 0,001**	43.19 ± 6.71	41.15 ± 38.17	0.125
**Tolerance (minute)**	8.13 ± 2.93	12.75 ± 2.64	**< 0,001**	7.88 ± 2.58	8.73 ± 2.85	0.116
**VAS (mm)**	28.58 ± 16.30	56.98 ± 22.09	**< 0,001**	45.86 ± 19.77	54.29 ± 20.87	**0.031**
**OSI**	0.45 ± 0.21	0.35 ± 0.28	**0.043**	0.50 ± 0.27	0.49 ± 0.26	0.561
**FBT**	10.19 ± 1.50	9.51 ± 1.99	**0,021**	10.12 ± 2.57	9.85 ± 1.92	0.158

*VRSQ* Virtual reality sickness questionnaire: *VAS* Visual analogue scale: *OSI* Overall stability index: *FBT* Flamingo balance test: Paired sample t-test, *p* < 0.05

There was a statistically significant difference between group x time interaction as determined by pre-test adjusted ANCOVA for VRSQ total (F(1,118) = 102.446, *p* < 0.001), VRSQ oculomotor (F(1,118) = 97.060, *p* < 0.001), VRSQ discomfort (F(1,118) = 105.910, *p* < 0.001), OSI (F(1,118) = 6.074, *p* = 0.015), and FBT scores (F(1,118) = 17.429, *p* < 0.001), along with an increase in tolerance time (F(1,118) = 63.916, *p* < 0.001) and VAS scores (F(1,118) = 26.671, *p* < 0.001) (Table 3.).

**Table 3:** Pre-test adjusted comparison of post-test scores between the groups

	Intervention (*n* = 60)	Placebo Control (*n* = 61)			
	X ± SD	X ± SD	F	*p*	ηp^2^
**VRSQ Total**	46.23 ± 16.79	74.24 ± 13.69	102.446	**< 0,001**	0.465
**VRSQ Oculomotor**	21.15 ± 7.98	33.82 ± 6.17	97.060	**< 0,001**	0.451
**VRSQ Discomfort**	25.08 ± 8.93	41.15 ± 8.17	105.910	**< 0,001**	0.473
**Tolerance (minute)**	12.75 ± 2.64	8.73 ± 2.85	63.916	**< 0,001**	0.351
**VAS (mm)**	45.86 ± 19.77	28.58 ± 16.30	26.671	**< 0,001**	0.184
**OSI**	0.49 ± 0.26	0.35 ± 0.28	6.074	**0.015**	0.049
**FBT**	0.64 ± 0.28	0.44 ± 0.32	17.429	**< 0,001**	0.129

*VRSQ* Virtual reality sickness questionnaire: *VAS* Visual analogue scale: *OSI* Overall stability index: *FBT* Flamingo balance test: ηp^2^ Partial ETA square: *F* ANCOVA test: *CI* Confidence Interval *p* < 0.05

Spearman correlation analysis was conducted to investigate the relationship between VRSQ and Titmus. There was a strong, negative significant correlation between the VRSQ and Titmus test scores (r = − 0.732, *n* = 121, *p* < 0.001).

## Discussion

The present study demonstrated that 4 weeks of combined balance therapy is effective in reducing motion sickness symptoms, increasing the duration of virtual reality usage, and enhancing user enjoyment in healthy adults with motion sickness.

According to studies, demographic data especially age has a major impact on the occurrence of motion sickness [30]. Additionally, it has been reported that motion sickness symptoms increase with the duration of application. However, adaptation to the VR environment is required in the initial use, and symptoms tend to decrease after 15–20 minutes of adaptation [31]. In the present study, baseline values of participants were similar in both groups, as they were separated according to their mental rotation skills, lateralization discrimination levels, age, gender, and Titmus scores. Also, all participants attended a session to familiarize the virtual environment.

The incorporation of VR applications into education presents numerous benefits. Nonetheless, despite these advantages, publications have reported limitations in the widespread adoption of VR as a general educational tool, primarily due to issues related to motion sickness [8, 17, 32]. Motion sickness produces many different symptoms. Among these, general discomfort and ocular symptoms are the most prominent [18]. Due to these symptoms, users’ tolerance and usage time of VR devices decreases significantly [33]. In the present study, similarly, 18.49% of the participants examined for eligibility had symptoms of motion sickness and were included in to study. The total motion sickness symptom scores showed a significant decrease in the intervention group compared to the control group. Furthermore, both the oculomotor and discomfort sub-parameters of VRSQ decreased after 4 weeks, signifying the effectiveness of the treatment program and implying that the duration of application was sufficient. Within the scope of our study, we examined the effectiveness of balance training on motion sickness. To the best of our knowledge, there is no information in the literature regarding rehabilitation protocols for virtual reality-induced motion sickness. Therefore, we were unable to compare the results of our study to those of other literature.

In the literature, it is reported that the duration of VR education sessions varies from 10 minutes to 45 minutes [34]. While the extended durations mostly include operation simulations, regional anatomy training was given in shorter durations [35]. The severity of reported symptoms and duration of experienced symptoms increases with prolonged usage [36]. Therefore, the duration of tolerance of the virtual environment is as important as the manifestation of symptoms caused by the virtual environment. In the present study, it was found that the tolerance duration within the VR environment increased by 49.6% compared to the pretest scores in the intervention group, while the control group increased by 11.2%. Increased tolerance to VR environment can be caused by reduced motion sickness symptoms in the intervention group. Especially VRSQ discomfort section potentially affects user enjoyment.

A notable aspect of VR is its capacity to offer a highly enjoyable experience. In a study conducted by Telner et al. 2010, 90.5% of participants self-reportedly concurred or strongly concurred with the statement, “I learn more when I have fun.” Enjoyment is deemed a crucial element, particularly in the context of case-based learning [37]. Several studies have reported that a significant majority of students express high levels of enjoyment when using VR for learning anatomy [38, 39]. Furthermore, self-direction, alongside enjoyment, is recognized as a pivotal factor contributing to the success of problem-based learning in medical education [40]. However, many of the symptoms that accompany motion sickness take the fun out of VR. In examining the impact of motion sickness on overall enjoyment, we found that the level of enjoyment from the virtual reality application increased in both groups. Nevertheless, upon comparing the two groups, it became evident that this increase was significant in favor of the intervention group. Increased enjoyment levels across both groups could be attributed to factors like growing familiarity with the virtual reality environment or improved user adaptation over time. The most likely explanation could be the placebo effect induced by the placebo exercises, as this increase occurred without any changes in motion sickness symptoms.

Impaired stability and increased sways can cause balance deficits due to impairments in proprioception and neuromuscular control [41]. The role of proprioception, the capacity to perceive one’s body’s position and movement, has not been thoroughly explored in the context of virtual reality. However, it plays a critical role in maintaining postural control, and the potential for heightened oscillations with reduced postural control to trigger motion sickness remains an intriguing area for future investigation [42]. Walter et al. 2019, report that when the complex task of body swaying is combined with the demanding movements of the virtual environment, and the sensory inputs inherent in these tasks do not align with real-world sensations, the risk of motion sickness increases significantly [43]. In addition to that, games with moving ground caused increased postural sways [44]. The finding of the present study revealed significant improvement in the stability index scores of participants in the intervention group. In contrast, there was no significant difference in post-test scores of OSI in the control group that received a placebo exercise program. These findings further support the hypothesis of the present study that motion sickness symptoms are related to sensory conflict. However, these improvements were below the Minimal Clinically Important Difference (MCID) scores reported by Yalfani et al. 2023, which were 0.38 for young women without a history of balance disorders [45]. All participants in the present study were healthy young adults who achieved nearly perfect scores in both balance and stability tests, leaving little room for improvement, which can explain the improvements below the MCID levels.

The literature reports that complex environments with a lot of interaction and moving background scenes create a sensory mismatch and cause falls [46]. Previous studies also reported that full immersive virtual reality affects the static balance [44, 47]. In the present study, static balance significantly improved in the intervention group compared to the control group. Both groups initially demonstrated good static balance (scoring 7 and above) and had no previous history of balance problems, the small difference between the pre-test and post-test scores was sufficient to demonstrate the effectiveness of the balance exercises. Although the change was statistically significant, the amount of change was below the MCID level (minimum 12% change from baseline [48]).

An unexpected discovery of our study was the strong negative correlation between motion sickness and stereoacuity test scores. Since the Titmus test measures stereoacuity, which refers to the ability to perceive depth and spatial relationships through binocular vision [49], this correlation hints at the possibility that individuals with greater stereoacuity may encounter fewer motion sickness symptoms. However, to truly comprehend the mechanisms at play in this relationship, further research is needed.

### Limitations

In this study, we used a combined intervention; therefore, we could not compare each section of the treatment protocol (proprioceptive, balance, and vestibular training) individually for motion sickness. Typically, anatomical education software and simulators do not involve acceleration, deceleration, and rotational movements. Therefore, the tolerance duration for educational software is expected to be significantly longer than what was observed in this study. Another limitation of the study was that it was conducted only on healthy young individuals.

## Conclusions

Combined balance training has been explored as a potential strategy for reducing motion sickness symptoms and found effective in both reducing motion sickness and increasing both overall enjoyment and usage time in virtual reality. The notable enhancements observed across a range of outcome measures, coupled with the substantial effect size, imply that incorporating such training could hold significant promise for improving the usability of VR in both educational settings and commercial fields. Further research may provide additional insights into the long-term effects and generalizability of the combined rehabilitation approach in diverse populations suffering from motion sickness in the virtual environment. Our results contribute information to the field of motion sickness management, potentially guiding future interventions and examining the effects on different patient groups, especially on individuals with balance problems, which will provide more detailed information to the literature.

## Data Availability

The datasets used and analyzed during the current study are available from the corresponding author on request.

## Abbreviations

FBT: Flamingo balance test
MS: Motion sickness
VRSQ: Virtual reality sickness questionnaire
VAS: Visual analogue scale

## References

[1] Radianti J, Majchrzak TA, Fromm J, Wohlgenannt I (2020). A systematic review of immersive virtual reality applications for higher education: design elements, lessons learned, and research agenda. Comput Educ..

[2] Cipresso P, Giglioli IAC, Raya MA, Riva G. The past, present, and future of virtual and augmented reality research: a network and cluster analysis of the literature. Front Psychol. 2018;2086 10.3389/fpsyg.2018.02086 PMC6232426 30459681

[3] Ryan GV, Callaghan S, Rafferty A, Higgins MF, Mangina E, McAuliffe F (2022). Learning outcomes of immersive technologies in health care student education: systematic review of the literature. J Med Internet Res..

[4] Kurul R, Ögün MN, Neriman Narin A, Avci Ş, Yazgan B (2020). An alternative method for anatomy training: immersive virtual reality. Anat Sci Educ..

[5] Suh A, Prophet J (2018). The state of immersive technology research: a literature analysis. Comput Hum Behav..

[6] Lie SS, Helle N, Sletteland NV, Vikman MD, Bonsaksen T (2022). Implementation of virtual reality in health professional higher education: protocol for a scoping review. JMIR Res Protoc..

[7] Chattha UA, Janjua UI, Anwar F, Madni TM, Cheema MF, Janjua SI (2020). Motion sickness in virtual reality: an empirical evaluation. IEEE Access..

[8] Kennedy RS, Fowlkes JE, Lilienthal MG (1993). Postural and performance changes following exposures to flight simulators. Aviat Space Environ Med..

[9] Cobb SV, Nichols S, Ramsey A, Wilson JR (1999). Virtual reality-induced symptoms and effects (VRISE). Presence: Teleoperat Virt Environ..

[10] Bronstein AM, Golding JF, Gresty MA, editors. Vertigo and dizziness from environmental motion: visual vertigo, motion sickness, and drivers' disorientation. Semin Neurol. 2013;33(03):219-30. 10.1055/s-0033-1354602 24057825

[11] Keshavarz B, Golding JF (2022). Motion sickness: current concepts and management. Curr Opin Neurol..

[12] Bles W, Bos JE, De Graaf B, Groen E, Wertheim AH (1998). Motion sickness: only one provocative conflict?. Brain Res Bull..

[13] LaViola JJ (2000). A discussion of cybersickness in virtual environments. ACM SIGCHI Bull..

[14] Bruck S, Watters PA (2009). Cybersickness and anxiety during simulated motion: implications for VRET. Annu Rev Cyberther Telemed..

[15] Golding JF, Gresty MA (2015). Pathophysiology and treatment of motion sickness. Curr Opin Neurol..

[16] Oman CM, Cullen KE (2014). Brainstem processing of vestibular sensory exafference: implications for motion sickness etiology. Exp Brain Res..

[17] Saredakis D, Szpak A, Birckhead B, Keage HA, Rizzo A, Loetscher T (2020). Factors associated with virtual reality sickness in head-mounted displays: a systematic review and meta-analysis. Front Hum Neurosci..

[18] Kim HK, Park J, Choi Y, Choe M (2018). Virtual reality sickness questionnaire (VRSQ): motion sickness measurement index in a virtual reality environment. Appl Ergon..

[19] Zhang LL, Wang JQ, Qi RR, Pan LL, Li M, Cai YL (2016). Motion sickness: current knowledge and recent advance. CNS neurosci therapeut..

[20] Berti S, Keshavarz B (2020). Neuropsychological approaches to visually-induced vection: an overview and evaluation of neuroimaging and neurophysiological studies. Multisens Res..

[21] Kim J, Oh H, Kim W, Choi S, Son W, Lee S (2020). A deep motion sickness predictor induced by visual stimuli in virtual reality. IEEE Transact Neural Netw Learn Syst..

[22] Gallagher M, Ferrè ER (2018). Cybersickness: a multisensory integration perspective. Multisens Res..

[23] Kiryu T, So RH (2007). Sensation of presence and cybersickness in applications of virtual reality for advanced rehabilitation. J neuroeng rehab..

[24] Yoon SY, Mann EL (2017). Exploring the spatial ability of undergraduate students: association with gender, STEM majors, and gifted program membership. Gifted Child Quart..

[25] Mazloumi Gavgani A, Hodgson DM, Nalivaiko E (2017). Effects of visual flow direction on signs and symptoms of cybersickness. PLoS One..

[26] Sevinc V, Berkman MI (2020). Psychometric evaluation of simulator sickness questionnaire and its variants as a measure of cybersickness in consumer virtual environments. Appl Ergon..

[27] Krkeljas Z (2018). Comparison of jump-landing protocols with Biodex balance system as measures of dynamic postural stability in athletes. Sports biomech..

[28] Sember V, Grošelj J, Pajek M. Balance Tests in Pre-Adolescent Children: Retest Reliability, Construct Validity, and Relative Ability. Int J Environ Res Public Health. 2020;17(15):5474. 10.3390/ijerph17155474 PMC7432309 32751279

[29] Kranti PB. A study to associate the flamingo test and the stork test in measuring static balance on healthy adults. Foot Ankle Online J. 2015;8(4)

[30] Dilanchian AT, Andringa R, Boot WR (2021). A pilot study exploring age differences in presence, workload, and cybersickness in the experience of immersive virtual reality environments. Fronti Virt Real..

[31] Risi D, Palmisano S (2019). Effects of postural stability, active control, exposure duration and repeated exposures on HMD induced cybersickness. Displays..

[32] Stanney KM, Graeber DA, Kennedy RS (2005). Virtual environment usage protocols. Handbook of standards and guidelines in ergonomics and human factors.

[33] Hussain M, Park J, Kim HK, Lee Y, Park S (2021). Motion sickness indexes in augmented reality environment. ICIC Express Lett Part B: Appl..

[34] Zhao J, Xu X, Jiang H, Ding Y (2020). The effectiveness of virtual reality-based technology on anatomy teaching: a meta-analysis of randomized controlled studies. BMC med educ..

[35] Uruthiralingam U, Rea PM (2020). Augmented and virtual reality in anatomical education–a systematic review. Biomed Visual..

[36] Koslucher F, Haaland E, Malsch A, Webeler J, Stoffregen TA (2015). Sex differences in the incidence of motion sickness induced by linear visual oscillation. Aerospace med human perform..

[37] Telner D, Bujas-Bobanovic M, Chan D, Chester B, Marlow B, Meuser J (2010). Game-based versus traditional case-based learning: comparing effectiveness in stroke continuing medical education. Can Fam Phys..

[38] Moro C, Štromberga Z, Raikos A, Stirling A (2017). The effectiveness of virtual and augmented reality in health sciences and medical anatomy. Anat Sci Educ..

[39] Maggio MP, Hariton-Gross K, Gluch J (2012). The use of independent, interactive media for education in dental morphology. J Dent Educ..

[40] Niehorster DC, Li L, Lappe M. The accuracy and precision of position and orientation tracking in the HTC vive virtual reality system for scientific research i-Perception. 2017;8(3):2041669517708205. 10.1177/2041669517708205 PMC5439658 28567271

[41] Terada M, Bowker S, Thomas AC, Pietrosimone B, Hiller CE, Rice MS, Gribble PA (2015). Alterations in stride-to-stride variability during walking in individuals with chronic ankle instability. Hum Mov Sci..

[42] Proske U, Gandevia SC. The proprioceptive senses: their roles in signaling body shape, body position and movement, and muscle force. Physiol Rev. 2012;92(4):1651-97. 10.1152/physrev.00048.2011 23073629

[43] Walter HJ, Li R, Munafo J, Curry C, Peterson N, Stoffregen TA (2019). Unstable coupling of body sway with imposed motion precedes visually induced motion sickness. Hum Mov Sci..

[44] Park S, Lee G (2020). Full-immersion virtual reality: adverse effects related to static balance. Neurosci Lett..

[45] Yalfani A, Bigdeli N, Gandomi F (2023). Comparing the effects of suspension and isometric-isotonic training on postural stability, lumbopelvic control, and proprioception in women with diastasis recti abdominis: a randomized, single-blinded, controlled trial. Physiother Theor Pract..

[46] RH So, WT Lo. Cybersickness: an experimental study to isolate the effects of rotational scene oscillations. Proceedings IEEE Virtual Reality (Cat No 99CB36316). 1999;237-24.

[47] Lo W, So RH (2001). Cybersickness in the presence of scene rotational movements along different axes. Appl Ergon..

[48] Miguel-Etayo D, Gracia-Marco L, Ortega F, Intemann T, Foraita R, Lissner L (2014). Physical fitness reference standards in European children: the IDEFICS study. Int J Obes..

[49] Wright KW. Clinical optokinetic nystagmus asymmetry in treated esotropes. J Pediatr Ophthalmol Strabismus. 2013;33(3):153–55. 10.3928/0191-3913-19960501-06 8771516

